# CEO birth order and corporate social responsibility behaviors: The moderating effect of female sibling and age gap

**DOI:** 10.3389/fpsyg.2022.1003704

**Published:** 2022-11-03

**Authors:** Minna Zheng, Guangqian Ren, Sihong Wu, Zezhen Jiang

**Affiliations:** ^1^School of Economics and Management, Hebei University of Technology, Tianjin, China; ^2^Business School, Zhengzhou University, Zhengzhou, China; ^3^School of Humanity and Law, Henan Agricultural University, Zhengzhou, China

**Keywords:** CEO's early family experience, CEO birth order, corporate social responsibility behaviors, female sibling, age gap

## Abstract

Corporate social responsibility (CSR) is one of the most important business strategies which helps enterprises obtain competitive advantage and improve performance. Scholars have conducted many beneficial studies on the driving factors of CSR behaviors from the perspective of CEO traits, but rarely focus on the impact of the CEO's early family experiences. This study aims to fill this research gap by investigating the influence of CEO birth order on firms' CSR behaviors, and further exploring the possible moderating effects of the presence of a female sibling and the age gap between the CEO and the closest sibling. This study takes Chinese non-financial private listed companies from 2010 to 2017 as the research samples, and empirically tests the relationship between CEO birth order and a firm's CSR behaviors. The empirical results show that CEO birth order negatively influences corporate social responsibility behaviors, and this relationship would be weakened when the CEO has a female sibling or the age gap between CEO and the closest sibling is larger. This paper extends the research on personal family factors from the field of social psychology to the business field and finds a new driving factor of corporate social responsibility behavior from the perspective of the CEOs' early family factors.

## Introduction

Enterprises have to make differentiation strategies to better cope with market competition and provide social support to their stakeholders (Zhou et al., 2022). For example, some enterprises developed new social media technology and adopted online technology to meet the changing needs of stakeholders during the epidemic to reduce the economic losses under the crisis (Yu et al., 2022). More critically, the growing external stakeholder pressure has raised requirements higher for corporate social responsibility (Lu and Abeysekera, 2017). Generally, CSR is regarded as a more competitive strategy to promote firms' pro-environmental behaviors, and helps firms to obtain good reputations and enhance their relationships with stakeholders (Tang et al., 2021), thereby promoting firms' sustainable business performances (Mubeen et al., 2021). Hence, how to promote CSR strategy is of great importance in helping enterprises maintain sustainable development in the post-epidemic era.

The driving factors of corporate social responsibility (CSR) strategy have received wide attention from both academic and practical fields. Among them, executives' traits are an important dimension to explain the choice of CSR strategy. Extant studies mainly focus on executives' demographic characteristics, educational background, and working experiences on corporate social behaviors (McCarthy et al., 2017; Tang et al., 2018; Al-Shammari et al., 2019). Little attention has been paid to the impact of executives' early family experiences on their behaviors in the CEO suit. And the childhood family experiences may greatly affect individuals' cognitive formation, personal preferences, and behaviors. Birth order is a natural difference that would influence individuals' early family interactions, which may predict individuals' psychological behavior (Taubman-Ben-Ari, 2018), and persist for the longest duration during adulthood (Whiteman et al., 2011).

Previous research about birth order mainly involved the sibling rivalry perspective, and explored the impact of birth order on individuals' risk-taking behaviors, such as smoking behavior (Slomkowski et al., 2005) and driving style (Taubman-Ben-Ari, 2018). Meanwhile, a few studies show that executives' birth order may also influence the firm's risk-taking behaviors where they work (Campbell et al., 2019). For example, Zheng L. J. et al. (2021) proposes that founders' birth order positively affects firms' innovation activities, which is usually known as one of the risk-taking behaviors. However, few studies have paid attention to sibling prosocial behaviors in addition to sibling rivalry, such as sharing, compassion, and help, especially in the business context. Considering that corporate social responsibility (CSR) behavior is usually seen as a typical prosocial behavior, this paper attempts to examine how executives' birth order affects corporate CSR behavior by considering their family traits.

In order to answer the above question, this paper takes Chinese non-financial private listed companies from 2010 to 2017 as the research samples, and employs a fixed effect model of panel data to empirically test the relationship between CEO birth order and the firms' CSR behaviors. We also examine the moderating effects of the presence of a female sibling and the age gap between CEO and the closest sibling. The empirical results show that there is a significant negative relationship between CEO birth order and corporate CSR behaviors. The results of further studies suggest that the presence of a female sibling weakens the negative impact of CEO birth order on firms' CSR behaviors. And the relationship between CEO birth order and CSR behaviors would also be weakened when the sibling age gap is larger.

This paper mainly contributes to three aspects: First, it enriches the studies of corporate social responsibility by exploring a new driving factor of CSR behavior from the perspective of CEOs' family traits. This paper explores how CEO birth order influences firms' CSR behaviors, and provides a new explanation of corporate CSR behaviors from executives' early family domain. Second, this study extends the research on the moderators of CEO birth order and CSR behaviors. To be specific, we mainly examine the moderating effects of the presence of a female sibling and the age gap between the CEO and the closest sibling and find that both the presence of a female sibling and a greater age gap would weaken the relationship between CEO birth order and CSR behaviors. Third, this paper advances the birth order research from sibling rivalry to sibling prosocial aspects. Previous studies mainly analyze the sibling effect on executives' behaviors based on the sibling rivalry view, while this paper integrates sibling prosocial tendencies and sibling rivalries into the same framework and proposes that sibling interaction may also shape executives' prosocial recognition and prosocial behaviors at their jobs.

The research arrangement of this paper is as follows: The second part is the literature review and hypotheses. The next part proposes the data and methodology. The fourth part reports the empirical analysis results, and the last part is the research conclusion and discussion of this paper.

## Literature review and hypotheses

### Sibling affection: Associate birth order with prosocial behaviors

Sibling relationship is an important motivator in shaping children's social recognition and behavioral tendencies persisting into their adulthood. Sibling interaction is characterized by affection, companionship, sharing, and helping, so that positive interaction with siblings may be conducive to form young children's prosocial preferences and then prosocial behaviors (Hughes et al., 2018). Through continual sibling prosocial interaction, children tend to imitate their elder siblings or parents' behaviors (Dunn and Munn, 1986), which enables children to learn how to share, cooperate, and help each other. These behaviors are prone to provide a behavioral mode for prosocial behaviors with others.

Sibling differences determine how children perceive the affection, warmth, competition, and conflict between siblings, which typically differ in age. Such age differences suggest that the elder children are more likely to express prosocial tendencies to their younger siblings by sharing, helping, and caretaking. Generally, when parents are busy with work and do not have enough time and energy to take care of the younger children, the elder children naturally take the responsibility for the younger siblings (Salmon et al., 2016). In this case, the elder siblings adopt more other-regarding behaviors toward the younger siblings, such as affection, help, and sympathy (Recchia and Howe, 2009). The early family experience of caring for younger siblings in childhood makes earlier-born children more likely to consider the feelings of others with empathy and affection (Otterbring and Folwarczny, 2022), and promotes their self-regulation and prosocial behavior (Padilla-Walker et al., 2010). By contrast, later-born children are more likely to form a self-interest tendency and less other-regarding or prosocial preferences, because they are often taken care of by others (Campbell et al., 2019).

### Sibling rivalry: Associate birth order with prosocial behaviors

Sibling interaction might also be full of rivalries. Faced with sibling competition over family resources, children would try their best to show their own unique abilities and characteristics, so as to get special attention and treatment from parents and improve their ability to acquire family resources (Wang et al., 2009). Because children have individual differences, parents tend to adopt differential treatment and unequally allocate family resources according to their children's individual characteristics (Tucker et al., 2003). This differential treatment negatively affects the quality of interaction between siblings and reduces their prosocial tendency (Shanahan et al., 2008). Birth order is a natural difference that enables children to maximize family resources and parental investment in different ways (Blake, 1981), and would also influence children's attitude toward family members and others (Harper et al., 2016). Those early family sibling experiences determine individuals' behavioral decisions during childhood and thus the whole life span (Suitor and Pillemer, 2007).

Birth order greatly influences sibling rivalry. For earlier-born children, parents have enough time and energy to care for them, and the household resources would also be relatively sufficient. Under this circumstance, sibling rivalries over family resources are relatively weaker (Booth and Kee, 2009). Moreover, elder siblings usually have a stronger ability of competition for resources (Freese et al., 1999), thus they easily get more household resources (Hotz and Pantano, 2015) and involve less in sibling rivalries. However, the amount of family resources available to each child would gradually decrease with the increase of the sibling number (Zheng M. et al., 2021). Meanwhile, the competition and conflict for parents' attention and family resources may be more intensified (Weng et al., 2019). Therefore, later-born children have to compete for parents' attention, time, and household resources with their elder siblings (Whiteman et al., 2011). Later-born children tend to be more competitive and unfriendly, which in turn stimulates individuals' short-term self-interest and makes them pay more attention to their own interests, thereby leading to more risky behaviors (Menesini et al., 2010; Solmeyer et al., 2013), antisocial behavior (Buist, 2010; Ensor et al., 2010) and fewer prosocial behaviors (Kretschmer and Pike, 2010; Buist and Vermande, 2014).

### CEO birth order and corporate social responsibility behavior

Family factors, such as family size, play a crucial role in entrepreneurship performance (Ge et al., 2022). Birth order is an important factor in personal early family life, and may shape individuals' recognition formation and behavioral tendencies (Zheng M. et al., 2021). Based on sibling affection literature, earlier-born siblings tend to exhibit more prosocial behaviors, while later-born individuals are usually engaged in less prosocial behaviors (Hughes et al., 2018). Birth order shapes individual's prosocial or antisocial preferences, so that executives' birth order may be closely related to the social responsibility behaviors of the company where they work. Therefore, we propose that CEOs' birth order negatively affects their prosocial behaviors and consequently firms' CSR behaviors. According to sibling interaction research, earlier-born individuals usually have a higher sense of family responsibility. And they are more likely to care for their younger sibling(s) and sympathize with others through their other-regarding tendencies (Salmon et al., 2016). This childhood affection experience shapes earlier-born individuals prosocial preferences and enables them to have a stronger motivation to participate in prosocial activities (Otterbring and Folwarczny, 2022). These findings suggest that earlier-born CEOs have a greater tendency to adopt prosocial behaviors toward employees, the public, and other stakeholders, and may implement more CSR behaviors through their business decisions. By contrast, later-born CEOs are often attendee and have fewer family responsibilities, so they are prone to engage in less prosocial behaviors.

In terms of sibling rivalry literature, CEO birth order affects parents' investment and the allocation of family resources; this early experience of sibling interaction was internalized into the CEOs' prosocial or antisocial bias. Earlier-born CEOs suffer less sibling rivalries and take much more family responsibility, which helps to form CEOs' prosocial orientations. This prosocial orientation improves the CSR behaviors that CEOs take in their executive suits. On the other hand, later-born CEOs have to compete more for family resources with their elder siblings, so they tend to form a sense of self-interest to maximize their own interests and less other-regarding preferences to others. This early family experience shapes CEOs' short-term self-interests and weakens their prosocial preferences, which would also reduce their attention on corporate social responsibility behaviors in the companies they occupy.

To sum up, the companies with the earlier-born CEOs might implement more social responsibility behaviors than those with the later-born CEOs. Based on the above analysis, this paper proposes the following hypothesis:

**Hypothesis 1:** CEO birth order is negatively correlated to firms' CSR behaviors.

### Moderating effect of the presence of a female sibling

Prior studies in sociology posit that women usually exhibit much stronger other-regarding preferences than men (Andreoni and Vesterlund, 2001; Dufwenberg and Muren, 2006; DellaVigna et al., 2013). And women often show a greater willingness and responsibility to help others (Kamas et al., 2008; Willer et al., 2015). Research on feminine ethics in the business field also indicates that women entrepreneurs often attach more importance on household affairs (Ge et al., 2022), and women executives focus more on stakeholders' interests and working relationships. Moreover, female directors or executives pay more attention to corporate social responsibility (Post et al., 2011; Atif et al., 2020) and charitable donations (Einolf, 2011).

Sibling interaction is a major family experience before adulthood, so the prosocial tendencies of female siblings could easily affect other siblings. The other-regarding preferences of women would be internalized into other siblings' behavioral tendencies through the family sibling interaction. When a CEO has an elder or little sister, the female sibling's other-regarding preferences are more likely to increase the focal CEO's prosocial orientation. Therefore, the presence of a female sibling moderates the relationship between CEO birth order and firms' CSR behaviors mainly through improving CEOs' prosocial preferences in their early family life, and weakens the negative influence of CEO birth order on firms' CSR behaviors. Hence, we posit the following hypothesis:

**Hypothesis 2:** The relationship between CEO birth order and corporate social responsibility behaviors would be weakened when the focal CEO has a female sibling.

### Moderating effect of sibling age gap

Since CEO birth order shapes their behavior tendencies during childhood (Sulloway, 2009), and sibling rivalry is one of the key mechanisms behind birth order effects (Wan et al., 2021), it follows that the factors which influence sibling rivalry may inevitably influence birth order effects and individuals' behavioral preferences. Accordingly, we suppose that the negative effect of CEO birth order on corporate social responsibility behaviors would be strengthened when the sibling rivalry is greater. Instead, if an individual's early family experience had less sibling rivalries, the differential treatment generated by birth order might also accordingly reduce, thus the relationship between CEO birth order and CSR behaviors would also be weakened.

Relevant research has shown that age gap influences the extent of sibling rivalry (Sulloway and Zweigenhaft, 2010). A smaller age gap indicates that siblings have to compete more fiercely for the scarce family resources and parents' attention (Badger and Reddy, 2009). And the elder siblings are less likely to care for the younger siblings under the conditions of a smaller age gap. But when the age gap is larger, siblings may have less rivalries for family resources, and parents also have more time and attention for their children over a greater age space (De Haan, 2010). Moreover, it is much more likely for the elder siblings to support their younger sibling when the age gap is larger, and the later-born siblings may also easily exhibit affection for their elder siblings (Dunn and Munn, 1986).

Above all, a closer age gap intensifies sibling rivalry and makes siblings compete more for family resources and parents' time. In this case, there is less siblings' prosocial behaviors and more siblings' competition. Conversely, a larger age gap reduces sibling rivalry and increases siblings' other-regarding preferences by taking care of other siblings. It suggests that the negative effect of CEO birth order and corporate social responsibility behaviors would be weaker when there is a larger age gap between CEOs and the closest siblings, and stronger when the age gap is smaller. Then we assume the following hypothesis:

**Hypothesis 3:** The relationship between CEO birth order and corporate social responsibility behaviors would be weakened when the age gap between a CEO and the closest sibling is larger and strengthened when the age gap is smaller.

### Theoretical framework of research model

**Figure 1:**
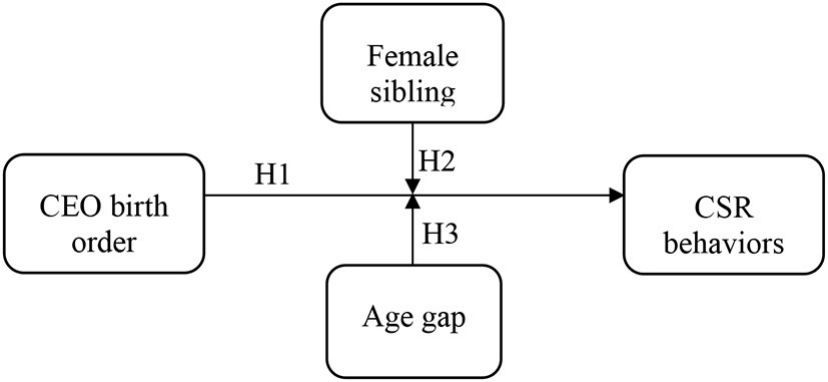
Theoretical framework incorporates key variables.

This study proposes a theoretical framework of the research model. This study investigates the relationship between CEO birth order and CSR behaviors of Chinese private firms, and further explores how the presence of a female sibling and age gap moderates the above relationship. Figure 1 describes the theoretical framework of the key factors. In this framework, CEO birth order is the independent variable, and CSR behavior is the dependent variable. Additionally, the presence of a female sibling and the sibling age gap are incorporated as the moderating variables. This study employs the fixed effects model of panel data to examine the impact of CEO birth order on CSR behaviors.

## Materials and methods

### Data and samples

In this paper, the Chinese A-share private listed companies on the Shanghai Stock Exchange and Shenzhen Stock Exchange from 2010 to 2017 were taken as data samples. Due to the fact that the corporate social responsibility of state-owned enterprises is largely subject to government administrative intervention, it is hard to investigate the relationship between CEO personal traits and CSR performance. Therefore, we chose Chinese private enterprises as the research samples. Then we excluded ST and ST^*^ samples, which refers to the companies that have been granted special treatment because of two consecutive years of losses, to avoid financial abnormality. The financial listed companies were also eliminated because of their high level of leverage. And samples with missing data of CEO birth order and other control variables were also excluded. Finally, we obtained 817 valid samples.

The data of corporate social responsibility (CSR) behaviors was obtained from the HeXun website. Considering that Huxun began to disclose the CSR Ratings of Chinese listed companies from 2010, we chose 2010 as the starting point of empirical samples. The original data of CEO siblings was obtained from the China Stock Market and Accounting Research (CSMAR) Database, which contains the detailed information about the gender and age of CEOs' relatives. Since the CSMAR database no longer discloses the executives' kinship data after 2017, this study sample period ends by 2017. Other control variables were all from the CSMAR Database except industry data from the WIND database. In order to avoid the influence of extreme values, all the continuous variables were winsorized at the 1% level.

### Variable definition

#### Dependent variable

##### Corporate social responsibility behaviors

According to Long et al. (2020), CSR behaviors are determined through the CSR ratings developed by HeXun, which has disclosed the social responsibility ratings of Chinese listed companies for many years and is usually used by Chinese scholars for CSR research. This CSR rating includes five aspects: responsibility for shareholders (30% weight), employees (15% weight), supply chain (15% weight), environment responsibility (20% weight), and social responsibility (20% weight). The HeXun CSR rating is mainly based on the corporate social responsibility reports and annual reports of Chinese listed companies, and could objectively and comprehensively measure CSR performance even for companies without disclosing the CSR report.

The RKS CSR rating mainly targets Chinese listed companies that have disclosed corporate social responsibility reports, but cannot assign a CSR Rating of listed companies that have not disclosed CSR reports. However, the proportion of social responsibility reports disclosed by listed private enterprises is relatively low in China, and only 128 sample companies with CEO sibling data disclosed CSR reports from 2010 to 2017. Hence, we used HeXun CSR Ratings to measure firms' CSR behaviors instead of RKS CSR Ratings to ensure a relatively large sample size and objective research conclusion.

#### Independent variable

##### CEO birth order

We first obtained the CEOs' names from the position information of Top Management Team (TMT) of listed companies in CSMAR Database. Then we further acquired the CEOs' sibling data from the TMT relatives database and dropped the samples without siblings. Based on the age of the CEOs and their siblings, the data of CEO birth order and the age gap between CEOs and their closest siblings were gleaned. Following extant studies, CEO birth order is ranked as the order CEOs were born. More precisely, the value of 1 was assigned to CEOs who are the first-born, and 2 for the second-born, etc. With reference to De Haan (2010) and Campbell et al. (2019), the CEO birth order was treated as a continuous variable in the regression models. In addition, we excluded the samples where CEOs were the only child.

#### Moderating variables

##### Presence of a female sibling

On the basis of the gender information of CEO siblings, we determined whether there was a female sibling for the focal CEOs. The presence of a female sibling was measured by a dummy variable that assigned a value of 1 when the focal CEO has a female sibling, otherwise 0.

##### Age gap

The variable of age gap between CEOs and their closest siblings was measured as the absolute difference value of the age between focal CEOs and their closest siblings (e.g., Buckles and Munnich, 2012). For example, when a CEO is first-born, the immediate second-born sibling is the closest sibling.

#### Control variables

With reference to prior studies on CSR behaviors, we introduced a list of CEO-level and firm-level control variables to avoid the regression bias. Relevant studies on birth order suggest that the number of siblings is inevitably related to birth order (Booth and Kee, 2009), so it was necessary to control CEOs' number of siblings in the regression models. Meanwhile, previous studies have shown that CEO personal traits may influence corporate social responsibility behaviors (Cronqvist and Yu, 2017; Hao et al., 2019). Thus, we controlled for CEO degree, CEO gender (1 for female CEOs and 0 for male CEOs), and CEO overseas background (coded as 1 when the CEO had overseas study or work experience, otherwise 0).

Second, we included several firm-level control variables into the regression models. Firm size was measured as the natural log of total assets. Financial leverage was calculated by the ratio of total liability to total assets. Return of Equity (ROE) was measured by the net income over average equity (Shaukat et al., 2016). Growth was measured as the growth rate of sales income. Additionally, we also controlled for governance-level variables. Board size (number of board directors), ratio of female directors (the proportion of female directors on board) (Landry et al., 2016), and ratio of directors with overseas background (the proportion of directors who have overseas study or work experience). The institutional shareholding ratio was measured as the number of institutional shareholders divided by the total number of shares (Dyck et al., 2019). H10 was calculated as the sum of the shares held by the top ten shareholders. Industry fixed effects and year fixed effects were all included in the regression models. Table 1 reports the detailed definition of all the variables.

**Table 1:** Variable definition.

**Variables**		**Symbol**	**Definition**
Dependent variable		CSR	CSR ratings score disclosed by HeXun Website
Independent variable		Birth order	the value of 1 is assigned for a first-born CEO, 2 for the second-born, etc
Moderating variable		Female sib	Code as 1 when a CEO has a female sibling, 0 otherwise
		Age gap	The age gap between CEO and the closest sibling
Control variable	CEO-level	Sib num	CEOs' number of siblings
		Degree	CEO' degree
		Gender	The gender of a CEO, 1 for male and 0 for female
		Overseas	Whether CEO has overseas study or work experience, “yes” marked 1, otherwise 0
	Corporate governance	BS	Number of board directors
		Fe ratio	Number of female directors/number of directors
		Ove ratio	Number of directors with overseas background/number of directors
	Firm-level	Inst	The number of institutional shareholders divided by the total number of shares
		H10	The sum of the squares held by the top ten shareholders
		ROE	Net income over average equity
		Size	The natural log of total assets
		Lev	Total liabilities/total assets
		Growth	the growth rate of sales income
		Industry	Industry dummy variable
		Year	Year dummy variable

### Models

According to the research hypothesis, we established Model (1) to test the impact of CEO birth order on firms' CSR behaviors. Model (2) and (3) were established to examine the moderating effects of the presence of a female sibling and age gap between a CEO and the closest sibling, respectively. Birthorder^*^Femalesib denotes the interaction term of CEO birth order and the dummy variable of the presence of a female sibling. Moreover, Birthorder^*^Agegap is the interaction term of CEO birth order and age gap between the focal CEOs and their closest siblings.


(1) $$\begin{array}{l} \begin{array}{lll} \text{CSR} & \text{=} & {\text{α+α}}_{\text{1}}^{\text{*}}{\text{Birthorder+α}}_{\text{i}}^{\text{*}}\text{Controls+Industry+Year} \end{array}\\ \;\begin{array}{ll} \text{+} & \text{ε} \end{array} \end{array}$$

(2) $$\begin{array}{l} \begin{array}{l} \begin{array}{lll} \text{CSR} & \text{=} & {\text{β+β}}_{\text{1}}^{\text{*}}{\text{Birthorder+β}}_{\text{2}}^{\text{*}}\text{Femalesib} \end{array}\\ \begin{array}{l} {\text{ + β}}_{\text{3}}^{\text{*}}{\text{Birthorder}}^{\text{*}}{\text{Femalesib+β}}_{\text{i}}^{\text{*}}\text{Controls+Industry }\\ \;\begin{array}{ll} \text{+} & \text{Year+ε} \end{array} \end{array} \end{array} \end{array}$$

(3) $$\begin{array}{l} \begin{array}{lll} \text{CSR} & \text{=} & {\text{γ+γ}}_{\text{1}}^{\text{*}}{\text{Birthorder+γ}}_{\text{2}}^{\text{*}}{\text{Agegap+γ}}_{\text{3}}^{\text{*}}{\text{Birthorder}}^{\text{*}}\text{Agegap} \end{array}\\ \begin{array}{ll} \text{ +} & {\text{γ}}_{\text{i}}^{\text{*}}\text{Controls+Industry+Year+ε} \end{array} \end{array}$$

Where ε is the residual error, α_i_ denotes the coefficient of control variables. Where Controls includes CEO degree, CEO gender, CEO overseas background, firm size, financial leverage, Return of Equity, Growth, Board Size, ratio of female directors, ratio of directors with an overseas background, Institutional shareholding ratio, and H10.

## Results analysis

### Descriptive statistics and correlation analysis

Table 2 reports the descriptive statistics of the main variables. The average CSR score of the sample companies is 24.85, the standard deviation is 13.77, indicating that the performance of different companies in terms of CSR behaviors varies greatly. The mean of CEO birth order is 1.53, and the standard deviation is 0.68, showing that there is a small gap in CEOs' birth order among different companies.

**Table 2:** Descriptive statistics.

**Variable**	***N***	**Mean**	**p50**	**SD**	**Range**	**Min**	**Max**
CSR	817	24.85	22.53	13.77	74.23	−1.32	72.91
Birth order	817	1.53	1.00	0.68	3.00	1.00	4.00
Sib num	817	1.27	1.00	0.53	4.00	1.00	5.00
Degree	778	3.60	3.00	1.48	6.00	1.00	7.00
Gender	817	0.94	1.00	0.23	1.00	0.00	1.00
Overseas	817	0.10	0.00	0.31	1.00	0.00	1.00
Size	817	21.48	21.43	0.87	4.47	19.87	24.34
Lev	817	0.31	0.29	0.18	0.79	0.03	0.82
ROE	817	0.09	0.09	0.07	0.44	−0.12	0.33
Growth	817	3.36	0.21	10.12	62.45	−4.95	57.50
BS	817	9.17	9.00	2.07	10.00	5.00	15.00
Fe ratio	817	0.17	0.14	0.13	0.54	0.00	0.54
Ove ratio	817	0.11	0.10	0.12	0.54	0.00	0.54
Inst	768	24.88	16.89	22.77	80.44	0.02	80.45
H10	817	0.18	0.17	0.10	0.45	0.04	0.49

From the descriptive statistics of the control variables, the average sibling number of the focal CEOs is 1.27. The average degree of CEOs is 3.60, indicating that more than half of the CEOs have a bachelor degree or above. And 94% of the CEOs are male, and the proportion of female CEOs is very small. The percentage of female directors and directors with an overseas background on the board is 17 and 11%, respectively, indicating that the proportion of female directors is relatively low in the sample companies. The mean of H10 is only 0.18, which shows that there is still a high level of equity concentration. Descriptive statistics of all variables are shown in Table 2.

Table 3 reports the correlations and the variance inflation factor (VIF). The average VIFs is < 2.0, far below the threshold of 10, so there is no serious multicollinearity problem in the regression process.

**Table 3:** Correlations.

	**CSR**	**Birth order**	**Sib num**	**Degree**	**Gender**	**Overseas**	**BS**	**Fe ratio**	**Ove ratio**	**Inst**	**H10**	**Size**	**Lev**	**ROE**	**Growth**	**VIF**
CSR	1															
Birth order	0.026	1														1.25
Sib num	0.024	0.468***	1													1.33
Degree	0.101***	0.075**	0.122***	1												1.07
Gender	0.069**	0.003	0.074**	0.084**	1											1.06
Overseas	−0.027	−0.106***	−0.158***	0.135***	0.083**	1										1.21
BS	0.049	−0.092***	−0.046	0.054	0.051	0.104***	1									1.07
Fe ratio	0.024	−0.070**	0.01	−0.048	−0.147***	−0.115***	−0.100***	1								1.08
Ove ratio	0.101***	−0.051	−0.116***	0.055	0.068*	0.318***	0.044	0.007	1							1.24
Inst	0.264***	0.000	−0.02	−0.042	0.032	0.042	0.113***	−0.073**	0.087**	1						1.35
H10	0.069*	0.028	0.094***	−0.062*	−0.052	−0.084**	−0.089**	0.032	−0.070**	0.079**	1					1.10
Size	0.182***	−0.029	−0.01	0.028	0.049	0.033	0.149***	0.024	0.243***	0.441***	0.003	1				1.79
Lev	−0.132***	−0.018	−0.052	−0.094***	0.067*	0.006	0.012	−0.044	0.058*	0.170***	−0.008	0.496***	1			1.35
ROE	0.456***	−0.017	−0.047	0.018	0.045	−0.018	0.038	0.007	0.069**	0.232***	0.217***	0.180***	−0.01	1		1.28
Growth	0.000	0.048	0.112***	−0.003	−0.001	−0.02	−0.061*	−0.036	−0.059*	−0.117***	0.089**	−0.128***	−0.084**	0.252***	1	1.16

^*^, ^**^, and ^***^ refer to significance at 10, 5, and 1% level.

### Regression results analysis

Table 4 reports the multiple regression results of CEO birth order and the firms' CSR behaviors. Hypothesis 1 assumes that CEO birth order is negatively correlated to CSR behaviors. The results of Model 1 show that the estimated coefficient between CEO birth order and CSR is −4.5781, and significant at the confidence level of 5% (*b* = −4.5781, *p* < 0.05). Therefore, CEO birth order has a negative and statistically significant impact on firms' CSR behaviors. That is, earlier-born CEOs pay more attention to CSR than later-born CEOs. This conclusion also holds in Model 2 and Model 3 even when including the interaction terms. Hypothesis 1 is thus confirmed.

**Table 4:** Regression results.

	**(1)**	**(2)**	**(3)**
**Variables**	**CSR**	**CSR**	**CSR**
Birth order	−4.5781**	−5.1229**	−7.7931***
	(−2.11)	(−2.44)	(−3.50)
Female sib		−15.3035***	
		(−3.02)	
Female sib * Birth order		17.2039***	
		(3.47)	
Age gap			−1.9840**
			(−2.12)
Age gap * Birth order			1.0217*
			(1.83)
Sib num	6.9376***	6.3234***	6.9203***
	(3.89)	(3.86)	(3.98)
Degree	−0.5145	−0.6427	−0.4246
	(−0.75)	(−0.91)	(−0.58)
Gender	−3.5711	−16.6446***	−5.9791
	(−0.87)	(−4.75)	(−1.56)
Overseas	−7.4864**	−7.4206**	−8.2379**
	(−2.05)	(−2.05)	(−2.02)
ROE	75.1301***	74.9032***	75.5313***
	(8.18)	(8.16)	(8.24)
Size	2.3201	2.2960	2.1775
	(1.04)	(1.03)	(0.97)
Lev	−5.4126	−5.7966	−4.6132
	(−1.06)	(−1.12)	(−0.88)
Growth	−0.1176***	−0.1188***	−0.1199***
	(−4.37)	(−4.38)	(−4.42)
BS	−0.2530	−0.2713	−0.2320
	(−0.77)	(−0.82)	(−0.71)
Fe ratio	18.6174**	19.3041**	17.8789**
	(2.17)	(2.22)	(2.07)
Ove ratio	−7.1997	−7.6099	−7.3347
	(−1.16)	(−1.22)	(−1.19)
Inst	0.0317	0.0312	0.0326
	(1.50)	(1.46)	(1.54)
H10	8.2985	8.8376	8.7674
	(0.71)	(0.75)	(0.75)
Industry	Yes	Yes	Yes
Year	Yes	Yes	Yes
Constant	−28.5580	−16.5185	−16.9378
	(−0.61)	(−0.35)	(−0.34)
Observations	733	733	733
Number of code	220	220	220
Adjusted *R*-squared	0.2597	0.2598	0.2612

^*^, ^**^, and ^***^ refer to significance at 10, 5, and 1% level. t-values are in parentheses.

In Hypothesis 2, this study predicts that the presence of a female sibling would weaken the negative relationship between CEO birth order and CSR behaviors. The results of Model 2 report that the estimated coefficient of the interaction term of the dummy variable of the presence of a female sibling and CEO birth order is 17.2039, and significant at the 1% level (*b* = 17.2039, *p* < 0.01). The above results indicate that the presence of a female sibling significantly weakens the negative impact of CEO birth order on firms' CSR behaviors. Hence, Hypothesis 2 is supported.

Hypothesis 3 theorizes the moderating effect of age gap on the relationship between CEO birth order and CSR behaviors. To examine this hypothesis, we introduce the interaction term of the age gap and CEO birth order in Model 3. The results of Table 3 suggest that age gap positively moderates the relationship between CEO birth order and CSR behaviors (*b* = 1.0217, *p* < 0.10). Specifically, the negative impact of CEO birth order on firms' CSR behaviors would be weakened when the sibling age gap is larger, and strengthened when the age gap is smaller. The above results statistically support Hypothesis 3.

**Figure 2:**
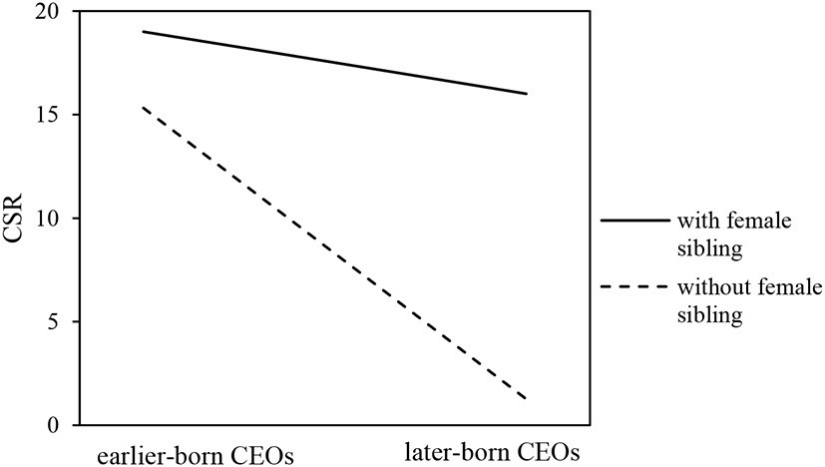
Moderating effect of the presence of a female sibling.

**Figure 3:**
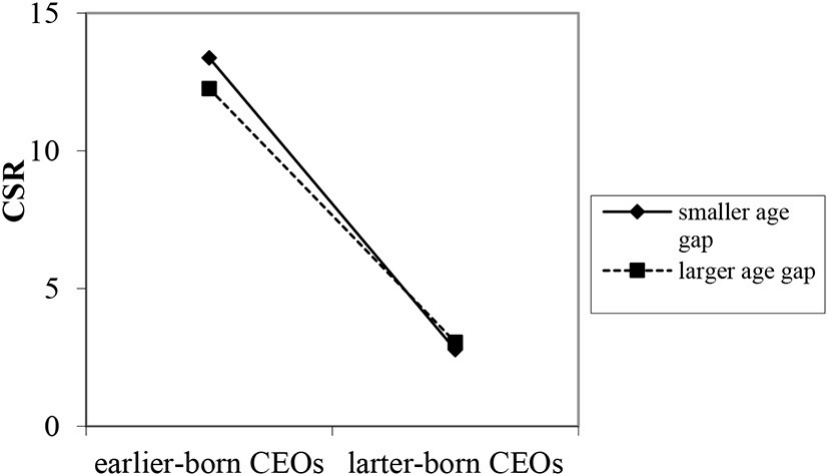
Moderating effect of sibling age gap.

With reference to Li et al. (2022), we further compare two figures to display the moderating effect of the presence of a female sibling and the age gap. Figure 2 shows the moderating role of the presence of a female sibling. It is easy to see that the presence of a female sibling would weaken the relationship between CEO birth order and corporate social responsibility behaviors. Figure 3 represents the moderating effect of sibling age gap. It indicates that the relationship between CEO birth order and CSR behaviors is weaker when the age gap between a CEO and the closest sibling is larger and stronger when the age gap is smaller.

### Robustness and endogeneity

According to Campbell et al. (2019), we treat CEO birth order as three categories: first-born, middle-born, and last-born, and then generate three dummy variables when CEOs are first-born, middle-born, and last-born, respectively. Column 1 of Table 5 reports the regression results including the dummy variable when CEOs are first-born, which shows that the first-born CEOs are positively influenced toward CSR behaviors (*b* = 6.9568, *p* < 0.05). Column 2 reports the result of the dummy variable of middle-born CEOs. The estimated coefficient of the dummy variable of middle-born CEOs is negative but not significant, which may be the result of the limited samples. Column 3 in Table 5 displays the impact on CSR behaviors when CEOs are last-born. The result indicates that the last-born CEOs are a significantly and negatively impacted toward CSR behaviors (*b* = −4.7025, *p* < 0.10). The above results suggest that later-born CEOs would exhibit less CSR than earlier-born CEOs.

**Table 5:** Robustness with birth order dummy variables.

	**(1)**	**(2)**	**(3)**
**Variables**	**CSR**	**CSR**	**CSR**
Dum_first	6.9568**		
	(2.13)		
Dum_middle		−0.3858	
		(−0.09)	
Dum_last			−4.7025*
			(−1.72)
Sib num	6.2586	6.0827	6.2044
	(0.97)	(0.93)	(0.96)
Degree	−0.2105	−0.1926	−0.4618
	(−0.31)	(−0.27)	(−0.66)
Gender	−7.5500	−5.9540	−2.8692
	(−1.28)	(−0.86)	(−0.47)
Overseas	−6.6522*	−7.8217**	−7.0535*
	(−1.68)	(−1.99)	(−1.79)
ROE	90.3607***	92.2907***	91.6389***
	(9.13)	(9.31)	(9.28)
Size	3.1804**	3.3181**	3.2469**
	(2.05)	(2.13)	(2.09)
Lev	−6.1716	−5.9536	−6.5662
	(−1.16)	(−1.11)	(−1.23)
Growth	−0.0501	−0.0478	−0.0532
	(−0.99)	(−0.94)	(−1.05)
BS	−0.2742	−0.2447	−0.2634
	(−1.02)	(−0.90)	(−0.97)
Fe ratio	18.4846***	16.0886**	18.0039***
	(2.75)	(2.41)	(2.68)
Ove ratio	−6.8688	−7.4336	−7.7180
	(−0.99)	(−1.06)	(−1.11)
Inst	0.0266	0.0288	0.0266
	(0.91)	(0.98)	(0.91)
H10	0.8068	1.7926	4.0956
	(0.06)	(0.13)	(0.29)
Industry	Yes	Yes	Yes
Year	Yes	Yes	Yes
Constant	−51.6337	−51.7491	−51.2151
	(−1.53)	(−1.51)	(-1.51)
Observations	733	733	733
Number of code	220	220	220
Adjusted *R*-squared	−0.0563	−0.0662	−0.0597

^*^, ^**^, and ^***^ refer to significance at 10, 5, and 1% level. t-values are in parentheses.

In order to test the robustness of the moderating effect of the presence of a female sibling, we further divided the sample companies into two groups according to whether the focal CEO has a female sibling or not, to implement the regression process. Table 6 reports the grouped regression results. In Column 1, the result shows that when the focal CEO has a female sibling, the negative impact of birth order on CSR behaviors is relatively weakened. However, the result in Column 2 indicates that CEO birth order has a much stronger influence on CSR behaviors when the focal CEO is without a female sibling (*b* = −4.6836, *p* < 0.05). Therefore, the negative relationship between CEO birth order and CSR behaviors is weakened when a CEO has a female sibling and strengthened when a CEO is without a female sibling.

**Table 6:** Robustness of a female sibling.

**Variables**	**Dum_fesib = 1**	**Dum_fesib = 0**
	**CSR**	**CSR**
Birth order	−0.9986	−4.6836**
	(−0.31)	(−2.20)
Sib num	–	5.6713***
	–	(3.56)
Degree	0.5335	−0.6067
	(0.30)	(−0.81)
Overseas	−5.6750	−9.6536**
	(−0.82)	(−2.50)
ROE	35.1570**	87.7599***
	(2.52)	(6.79)
Size	3.0263	2.8578
	(0.88)	(1.02)
Lev	6.7574	−6.3189
	(0.50)	(−1.04)
Growth	−0.0824**	−0.1362***
	(−2.05)	(−3.10)
BS	−0.6521*	−0.1359
	(−1.70)	(−0.33)
Fe ratio	29.5311**	17.0546
	(2.29)	(1.49)
Ove ratio	−8.6656	−6.3803
	(−0.76)	(−0.89)
Inst	0.0232	0.0354
	(0.77)	(1.42)
H10	−5.3584	18.2769
	(−0.34)	(1.17)
Industry	Yes	Yes
Year	Yes	Yes
Constant	−41.6787	−44.2679
	(−0.64)	(−0.74)
Observations	185	548
Number of code	64	159
Adjusted *R*-squared	0.1576	0.2829

^*^, ^**^, and ^***^ refer to significance at 10, 5, and 1% level. t-values are in parentheses.

Based on the study of Baer et al. (2005), we further used a discrete measurement of age gap to test the moderating effect of the closest sibling age gap. Specifically, we created a dummy variable and code as 1 when the age gap between a CEO and the closest sibling is more than 3 years. Column 1 of Table 7 reports the result including the dummy variable of the closest age gap, which shows that a larger age gap weakens the negative impact of CEO birth order on CSR behaviors (*b* = 5.7597, *p* < 0.01). The result also statistically supports Hypothesis 3. Moreover, we divided the samples into two groups on the basis of the age gap dummy variable to repeat the regression process of Model 1. The grouped results also indicate that a smaller age gap would strengthen the negative relationship between CEO birth order and CSR behaviors (*b* = −7.1490, *p* < 0.01).

**Table 7:** Robustness of age gap.

		**Dum_agegap = 1**	**Dum_agegap = 0**
**Variables**	**CSR**	**CSR**	**CSR**
Birth order	−6.1481***	−2.3854	−7.1490***
	(−3.42)	(−0.88)	(−3.22)
Dum_age gap	−13.3233**		–
	(−2.35)		
Dum_age gap *	5.7597**		−1.6661
Birth order	(2.01)		(−1.18)
Sib num	6.9445***	8.4267***	−15.8540***
	(3.97)	(2.89)	(−4.66)
Degree	−0.3826	0.8671	−1.3283
	(−0.51)	(1.58)	(−0.29)
Gender	−6.3106	−5.5161	85.3011***
	(−1.61)	(−1.53)	(6.44)
Overseas	−8.2978**	−17.6702***	−1.1183
	(−2.07)	(−5.65)	(−0.39)
ROE	76.0486***	66.7870***	5.7938
	(8.20)	(5.25)	(0.88)
Size	2.1435	5.9549*	−0.1016***
	(0.95)	(1.78)	(−2.85)
Lev	−4.5124	−17.2829**	−0.3874
	(−0.86)	(−2.15)	(−0.93)
M/B	−0.1206***	−0.1456***	11.2006
	(−4.43)	(−2.78)	(1.02)
Growth	−0.2285	−0.2954	−11.6294
	(−0.70)	(−0.69)	(−1.59)
BS	18.1924**	33.1415***	0.0227
	(2.14)	(2.66)	(0.73)
Fe ratio	−7.1534	−6.3524	7.0830
	(−1.16)	(−0.67)	(0.46)
Inst	0.0308	0.0626*	69.1510
	(1.47)	(1.88)	(1.10)
H10	9.1669	41.8737**	375
	(0.77)	(2.00)	109
Industry	Yes	Yes	Yes
Year	Yes	Yes	Yes
Constant	−18.5981	−114.9483	0.3226
	(−0.38)	(−1.59)	(0.92)
Observations	733	358	375
Number of code	220	114	109
Adjusted R-squared	0.2611	0.2417	0.3196

^*^, ^**^, and ^***^ refer to significance at 10, 5, and 1% level. t-values are in parentheses.

According to Weng et al. (2019), we used a two-stage Heckman selection model and two exogenous variables to tackle the possible endogeneity bias caused by sample selection. The first one was sex of first child. In Chinese traditional cultural context, when the first child is a girl, parents are more likely to have more than one child to ensure that there is a boy to maintain the family. Therefore, sex of first child may influence the number of siblings and then birth order. The second exogenous variable is the family planning policies. Weng et al. (2019) divided China's family planning policies into four phases based on the birth quota. Therefore, we marked the four phases as 0–3 corresponding to CEO birth year, to reflect the degree of government control for birth quota. Table 8 shows the results with Mills generated by the two exogenous variables, which are still in line with our main conclusion.

**Table 8:** Heckman two-stage results.

	**(1)**	**(2)**	**(3)**
**Variables**	**CSR**	**CSR**	**CSR**
Mills	28.2629*	30.4241*	33.5120*
	(1.69)	(1.77)	(1.97)
Birth order	−2.9341*	−3.4880**	−4.4149**
	(−1.82)	(−2.38)	(−2.47)
Female sib		−16.6464***	
		(−3.20)	
Female sib * Birth order		28.9200***	
		(3.74)	
Age gap			−1.3907
			(−1.46)
Age gap * Birth order			0.4935
			(0.86)
Sib num	−1.4316	−2.4066	−1.4303
	(−0.37)	(−0.62)	(−0.37)
Degree	−0.7586	−0.8012	−0.7817
	(−0.90)	(−0.95)	(−0.87)
Gender	−3.7095	−27.2194***	−4.8931
	(−0.76)	(−4.56)	(−0.91)
Overseas	−3.4804	−3.2835	−4.2418
	(−0.76)	(−0.73)	(−0.83)
ROE	82.9547***	82.2366***	82.6667***
	(8.36)	(8.28)	(8.34)
Size	2.6735	2.6453	2.5210
	(1.13)	(1.12)	(1.06)
Lev	−1.2868	−1.7659	−1.2131
	(−0.20)	(−0.28)	(−0.19)
Growth	−2.0542*	−2.1119*	−1.9746*
	(−1.78)	(−1.80)	(−1.72)
BS	−0.1519	−0.1865	−0.1432
	(−0.43)	(−0.52)	(−0.40)
Fe ratio	26.3332**	27.5067***	26.6336**
	(2.56)	(2.63)	(2.58)
Ove ratio	−12.8093*	−12.8900*	−12.8801*
	(−1.83)	(−1.83)	(−1.83)
Inst	0.0358	0.0319	0.0350
	(1.25)	(1.10)	(1.21)
H10	4.7848	5.6483	4.4687
	(0.42)	(0.51)	(0.39)
Industry	Yes	Yes	Yes
Year	Yes	Yes	Yes
Constant	−65.2247	−50.2421	−62.1835
	(−1.08)	(−0.82)	(−1.02)
Observations	595	595	595
Number of code	170	170	170
Adjusted *R*-squared	0.2481	0.2492	0.2467

^*^, ^**^, and ^***^ refer to significance at 10, 5, and 1% level. t-values are in parentheses.

## Conclusions and implications

### Conclusions and discussion

Enterprises are practicing CSR, business modes, and entrepreneurial networks with innovation and knowledge sharing to improve business performance (Rahmat et al., 2022; Zhou et al., 2022). Among the above activities, CSR is often considered as the basis of competitive advantages and an important way to increase firms' value (Tang et al., 2021). CEOs are highly correlated with firms' CSR activities (Mubeen et al., 2021). Therefore, this study aims to investigate the driving factors of firms' CSR behaviors from the CEO perspective, and explores the relationship between CEO birth order and corporate CSR behaviors of Chinese private firms, through the moderating role of the presence of a female sibling and sibling age gap. This research combines sibling prosocial tendencies and sibling rivalry into a whole framework, and extends the research of sibling effect from family perspectives to a business context.

This paper constructs a theoretical framework to explore how CEO birth order influences corporate social responsibility behaviors. The study takes Chinese A-share private listed companies from 2010 to 2017 as data samples, to empirically test the relationship between CEO birth order and firms' CSR behaviors. The empirical results show that there is a significant and negative relationship between CEO birth order and firms' CSR behaviors. In other words, earlier-born CEOs tend to implement more CSR than later-born CEOs, while the later-born CEOs are inclined to take less CSR behaviors. The findings of this paper are basically consistent with the previous literature of sibling prosocial behaviors and sibling rivalry (Zheng L. J. et al., 2021; Zheng M. et al., 2021). It suggests that earlier-born individuals are more likely to exhibit prosocial behaviors to their siblings and others. By contrast, later-born individuals are generally the ones being cared for, so they are more self-concerned and have less prosocial preferences.

This study further investigates the moderating role of the presence of a female sibling and sibling age gap on the relationship between CEO birth order and firms' CSR behaviors. The results show that the influence of CEO birth order on CSR behaviors will be weakened when the focal CEO has a female sibling. The above results support the view of female socialization, which proposes women usually have higher social preferences and tend to positively influence their family members' prosocial behaviors (Cronqvist and Yu, 2017). Moreover, the negative relationship between CEO birth order and firms' CSR behaviors would be weaker when there is a larger age gap between a CEO and the closest sibling, and stronger when the age gap is smaller. The conclusion indicates that a smaller age gap intensifies the sibling rivalries and reduces siblings' prosocial bias, which is primarily in line with the study of Campbell et al. (2019).

### Implications

#### Theoretical implication

This paper has three theoretical implications for the existing literature: First, the research on family sibling effect is extended from the field of social psychology to the business context. This paper enriches the studies on the influence of executives' early life experience on corporate strategic decision-making. The research on family sibling effect in the field of social psychology mainly focus on the influence of sibling effect on an individual's internal psychology or external behavior. As an individual, a CEO's early family life inevitably affects their cognitive formation and behavior preferences, which will be brought forward to the strategic decision of the enterprises they manage.

Second, this study enriches the research on the driving factors of CSR and finds a new driving factor of CSR behaviors. Existing research has explored the driving factors of firms' CSR behaviors from the perspective of CEO traits and adulthood experiences (McCarthy et al., 2017; Tang et al., 2018; Hegde and Mishra, 2019), but few studies focus on the influence of CEOs' early family life experiences on CSR. From the perspective of CEOs' early family traits, this paper investigates the influence of CEO birth order on the CSR behaviors of the company they serve in the adulthood. Our research shows that CEO birth order shapes their personal prosocial tendency by influencing the sibling rivalry and prosocial preferences, which directly influences the firms' CSR behaviors.

Third, this paper expands the upper echelon theory by examining the effect of executives' family traits and childhood experiences on corporate social responsibility behaviors. Many studies based on the upper echelon theory have focused on the impact of CEOs' demographic characteristics and work experience on CSR behaviors (McGuire et al., 2003; Deckop et al., 2006; Tang et al., 2015, 2018), neglecting the important role of early family experiences on CEOs' behavioral preference and corporate decision-making. From the perspective of CEOs' early family experiences, this paper studies how birth order affects corporate social responsibility behaviors by influencing CEOs' prosocial tendencies, which is conducive to a profound understanding of the influence of CEOs' early experiences on their business behaviors and decision-making.

#### Policy recommendations

There are also two main practical implications: First, it provides a further reference for listed companies that are concerned about corporate social responsibility to consider individuals' early family context when recruiting executives. For listed companies that pay attention to CSR, the number of siblings, birth order, and other early family environment should be taken into consideration when selecting CEOs, so as to ensure the effective performance of corporate social responsibility and maintain firms' sustainable development. Second, CEOs should be aware of the impact of birth order and other early family traits on their decision-making. Earlier-born CEOs tend to engage in more prosocial behaviors and take more appropriate social responsibility strategy. In contrast, later-born CEOs are more likely to adopt less CSR behaviors at their job. Therefore, CEOs need to acknowledge the association between birth order and firms CSR performance when making strategic decisions. Third, the study encourages enterprises to establish effective corporate governance structure and mechanisms to supervise the behaviors of executives and make corporate decisions free from the influence of executives' personal preferences. The absence of effective supervision mechanisms increases executives' opportunism, which enables executives to make decisions based on personal preferences rather than corporate interests. Hence, it is necessary to improve corporate supervision mechanisms through the optimization of corporate governance structure and governance mechanisms.

#### Research limitations

This study mainly has the following limitations: First, we theorized that CEO birth order influences firms' CSR behaviors through affecting sibling rivalry and shaping other-regarding preferences, but we cannot directly examine the birth order effect of the past sibling rivalry and family life experience. Although we further tested the hypothesis through moderators to provide additional evidence to our conclusion, there is still a need to explore a proper way to deeply investigate the internal mechanism behind CEO birth order effect. Second, we only chose Chinese private enterprises with CEO sibling data as research samples, there may be endogeneity problems especially caused by sample-selection bias. Although we have used a Heckman two-stage model to deal with the endogeneity problems, future studies are still needed to further investigate CEO sibling effect with more comprehensive samples. Third, this paper only studies the influence of CEOs' sibling effects on firms' CSR behaviors, but does not consider different situational contexts. Future research can further investigate the influence of regional economic development level, cultural factors, and other factors on CEOs' sibling effects and business behaviors. Fourth, due to the limitations of the research sample, this paper only uses the data from 2010 to 2017 for empirical analysis. Future research can further expand the research sample and examine the influence of situational factors, such as the COVID-19 epidemic.

## Data availability statement

The original contributions presented in the study are included in the article/supplementary material, further inquiries can be directed to the corresponding author/s.

## Author contributions

MZ provided the overall conceptual model and wrote the original manuscript. GR modified the manuscript and provided ideas and suggestions for revision. SW and ZJ provided supplementary data and analysis for the revision. All authors contributed to the article and approved the submitted version.

## Funding

This study was supported by Key Projects of the National Social Science Foundation of China (22AGL017), Youth Fund for Humanities and Social Sciences Research of the Ministry of Education (22YJC790082), Annual Program of Philosophy and Social Science Planning in Henan Province (2022BJJ115), and General Research Projects of Humanities and Social Sciences in Colleges and Universities in Henan Province (2021-ZZJH-366).

## Conflict of interest

The authors declare that the research was conducted in the absence of any commercial or financial relationships that could be construed as a potential conflict of interest.

## Publisher's note

All claims expressed in this article are solely those of the authors and do not necessarily represent those of their affiliated organizations, or those of the publisher, the editors and the reviewers. Any product that may be evaluated in this article, or claim that may be made by its manufacturer, is not guaranteed or endorsed by the publisher.
